# Supplementary material for: Structural basis for recognition of 53BP1 tandem Tudor domain by TIRR
Source: Nat Commun. 2018 May 29;9:2123. doi: 10.1038/s41467-018-04557-2 (PMC5974088; doi:10.1038/s41467-018-04557-2)
Supplement: Supplementary file 1 — Supplementary Information [file 41467_2018_4557_MOESM1_ESM.pdf]

Structural basis for recognition of 53BP1 tandem Tudor domain by TIRR

Dai *et al.*

Supplementary Figure 1

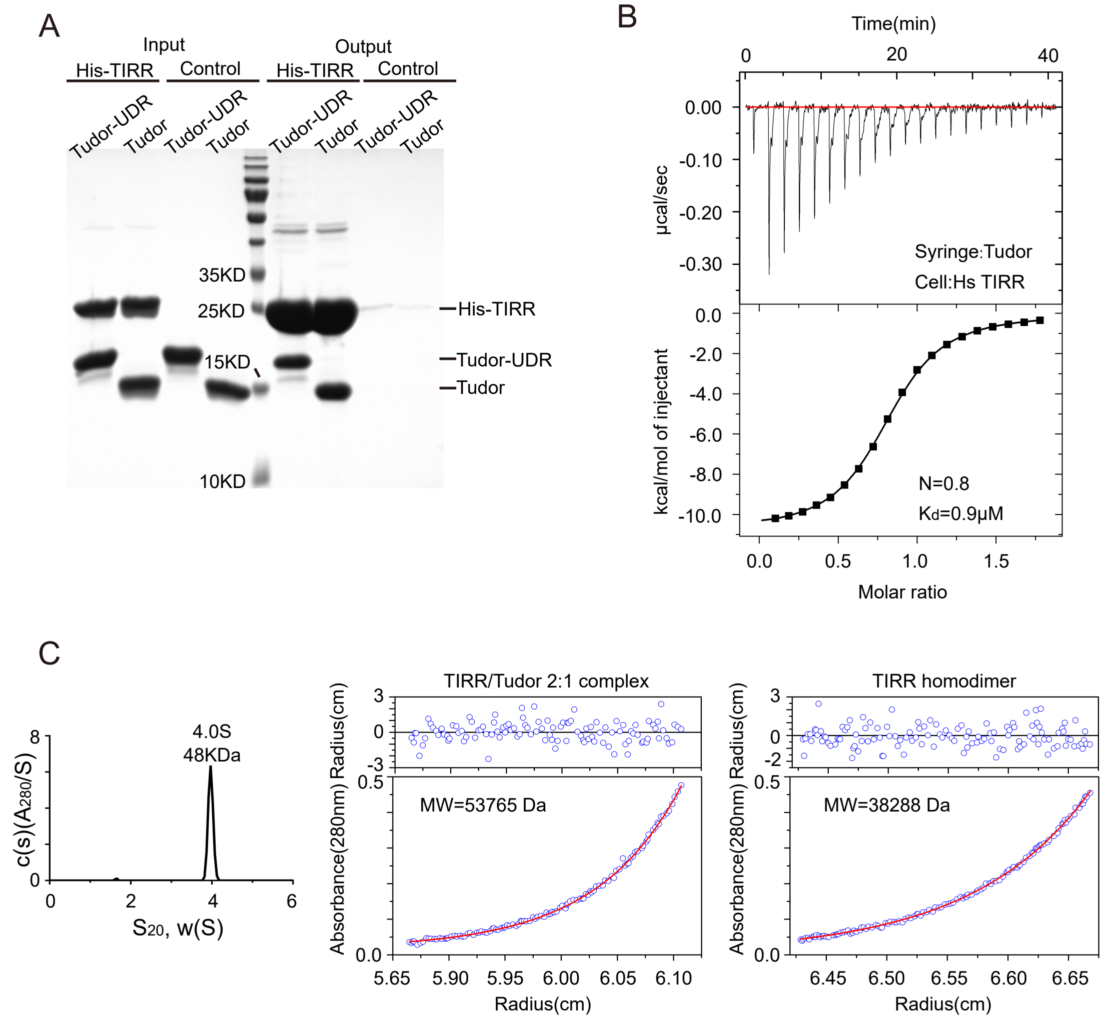

Supplementary Fig. 1. TIRR forms a stable complex with 53BP1 Tudor domain. **A.**Pull-down assay with His-tagged TIRR and 53BP1 Tudor and Tudor-UDR. **B.**ITC analysis of 53BP1 tandem Tudor domain interacting with the human TIRR.**C.**Analytical ultracentrifuge analysis of TIRR/53BP1 Tudor complex. Displayed are sedimentation velocity result (left) and sedimentation equilibrium result (middle) of TIRR/53BP1 Tudor complex premixed at 1:2 ratio. Sedimentation equilibrium result of TIRR homodimer is shown on the right. The sedimentation coefficient distributions c(s) are plotted in relative absorbance units A280 versus svedbergs (S).

Supplementary Figure 2

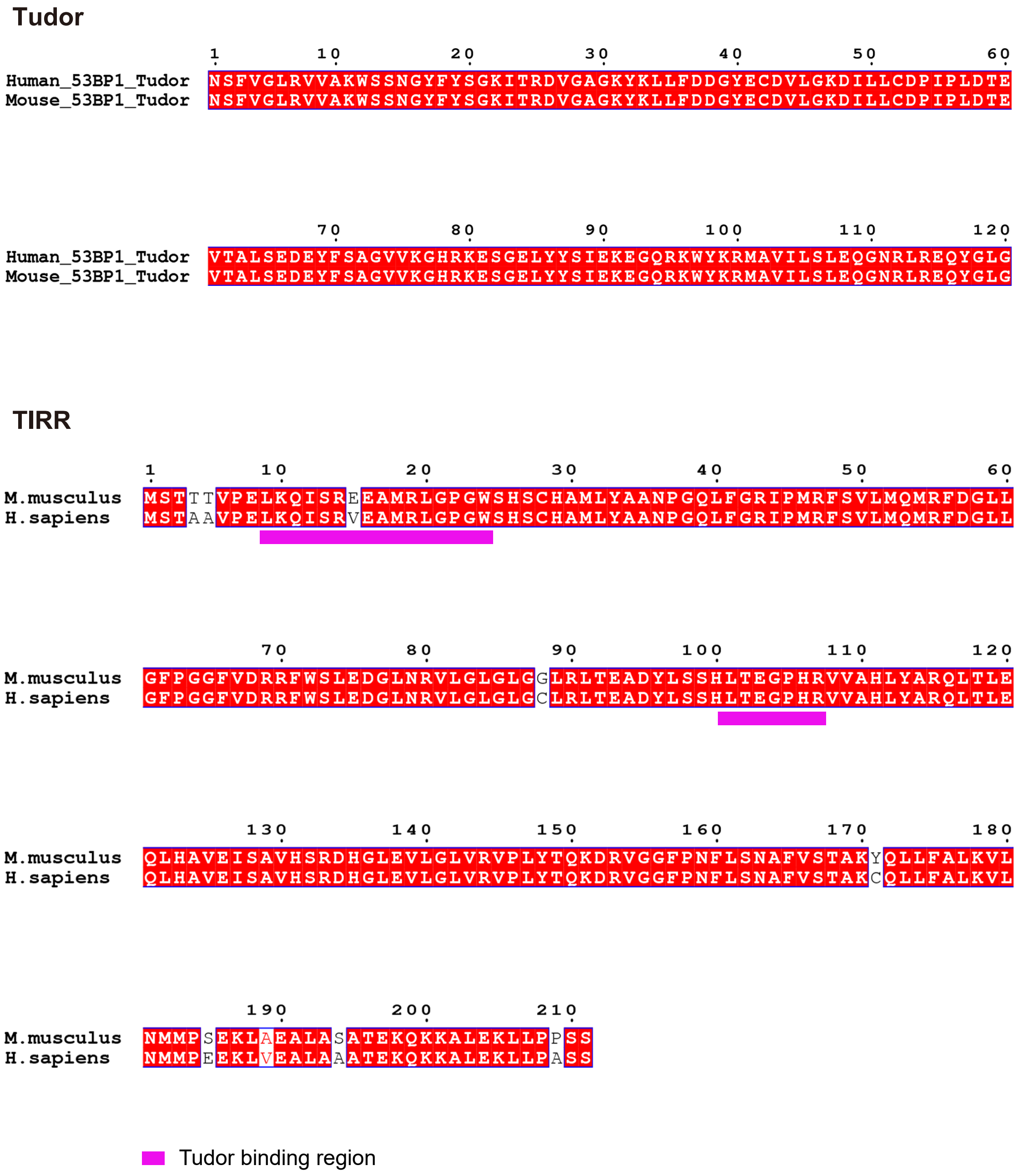

Supplementary Fig. 2. Sequence alignments of 53BP1Tudor domain and TIRR from human and mouse. Highlighted in cyan represent TIRR regions interacting with 53BP1 Tudor.

Supplementary Figure 3

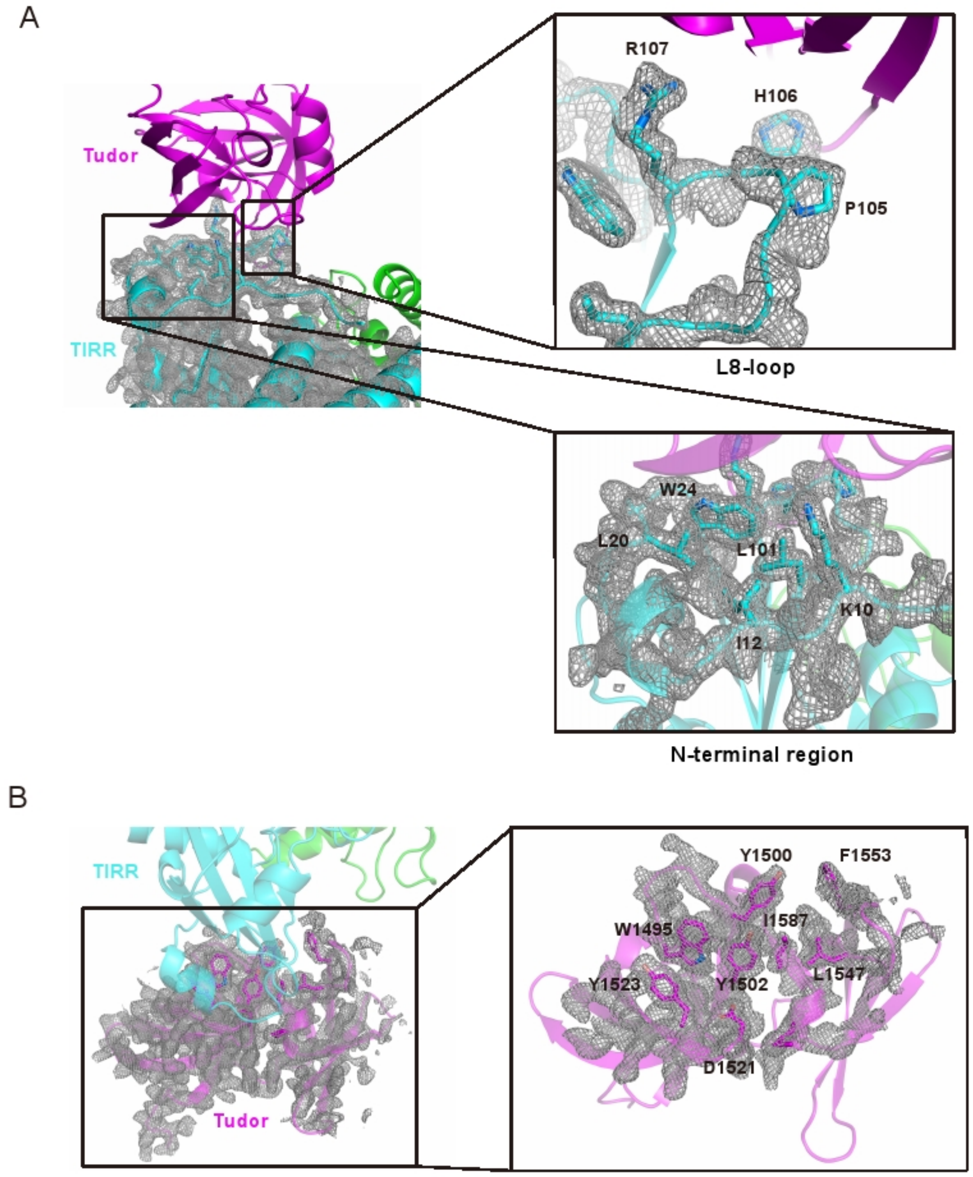

Supplementary Fig. 3. Electron density maps for interaction interfaces in 53BP1 and TIRR. The 2Fo-Fc omit map contoured at 1σ level (gray mesh,generated by PHENIX) displaying the full electron density for the interface are shown for structures of TIRR (**A**), and 53BP1 Tudor(**B**). The densities of interaction interface in Tudor, N-terminal region and L8 loop of TIRR are zoomed in pane.

Supplementary Figure 4

A

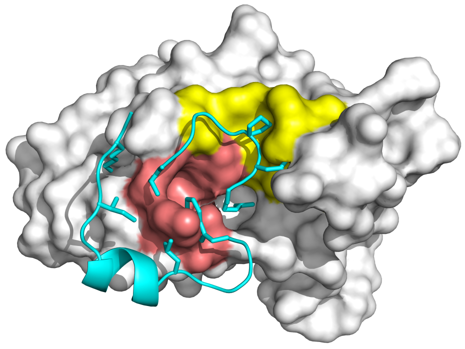

Tudor(H4K20me2)

B

Tudor domain	Binding affinity(K_d,μM)	
	*H4K20me2	TIRR
WT	19.7	0.9
W1495A	>1500	15.4
Y1500A	162.9	12.7
Y1502A	151	ND
D1521A	>1500	5.4
D1521R	None	ND
Y1523A	7.8	28.5
Y1523S	65.8	ND

*Data[1] from Botuyan, et al, *cell*, 2006
ND means Not Detected

Supplementary Fig. 4. Comparison of H4K20me2-bound 53BP1 Tudor with TIRR -bound 53BP1 Tudor reveals distinct binding modes. A. Surface presentation of H4K20me2-bound 53BP1 Tudor in superimposition with TIRR-bound 53BP1 Tudor. Highlighted in different colors are 53BP1 residues forming the aromatic cage (salmon) and other residues involved in H4K20me2 binding (yellow).TIRR residues presented in sticks (cyan) show steric hindrance with H4K20me2-bound 53BP1 Tudor.**B**.H4K20me2 and TIRR interact with 53BP1 Tudor mutants with different binding affinities.

Supplementary Figure 5

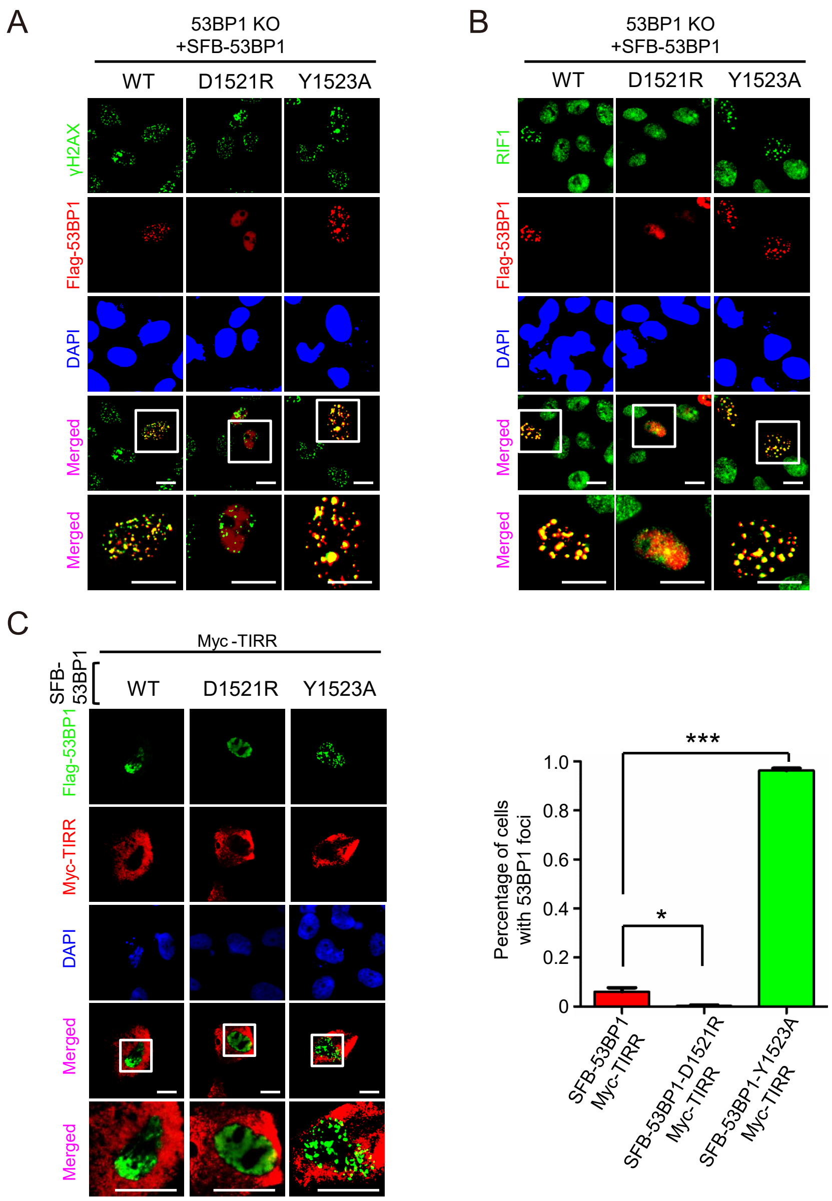

Supplementary Fig. 5. Critical residues within Tudor domain of 53BP1 participate in 53BP1-mediated DNA repair. (A,B) MCF10A derived 53BP1 knockout cells were transfected with plasmids encoding SFB-tagged wild-type (WT), D1521R or Y1523A construct followed by ionizing radiation. Four hours later cells were fixed and immunostained with anti-FLAG and anti-γH2AX (A) or anti-RIF1 (B).Scale bar represents 200um. **C.**MCF10A cells were transfected with plasmids encoding SFB-tagged 53BP1 wildtype (WT), D1521R or Y1523A construct along with Myc-tagged TIRR construct followed by ionizing radiation. Four hours later cells were fixed and immunostained with anti-FLAG and anti-Myc. FLAG-53BP1 foci in these cells were quantified (at least 400 cells were counted for each experiment; three independent experiments). Data are represented as the mean ± s.d.(n=3) *, $p < 0.05$; ***, $p < 0.001$.Scale bar represents 200um.

Supplementary Figure 6

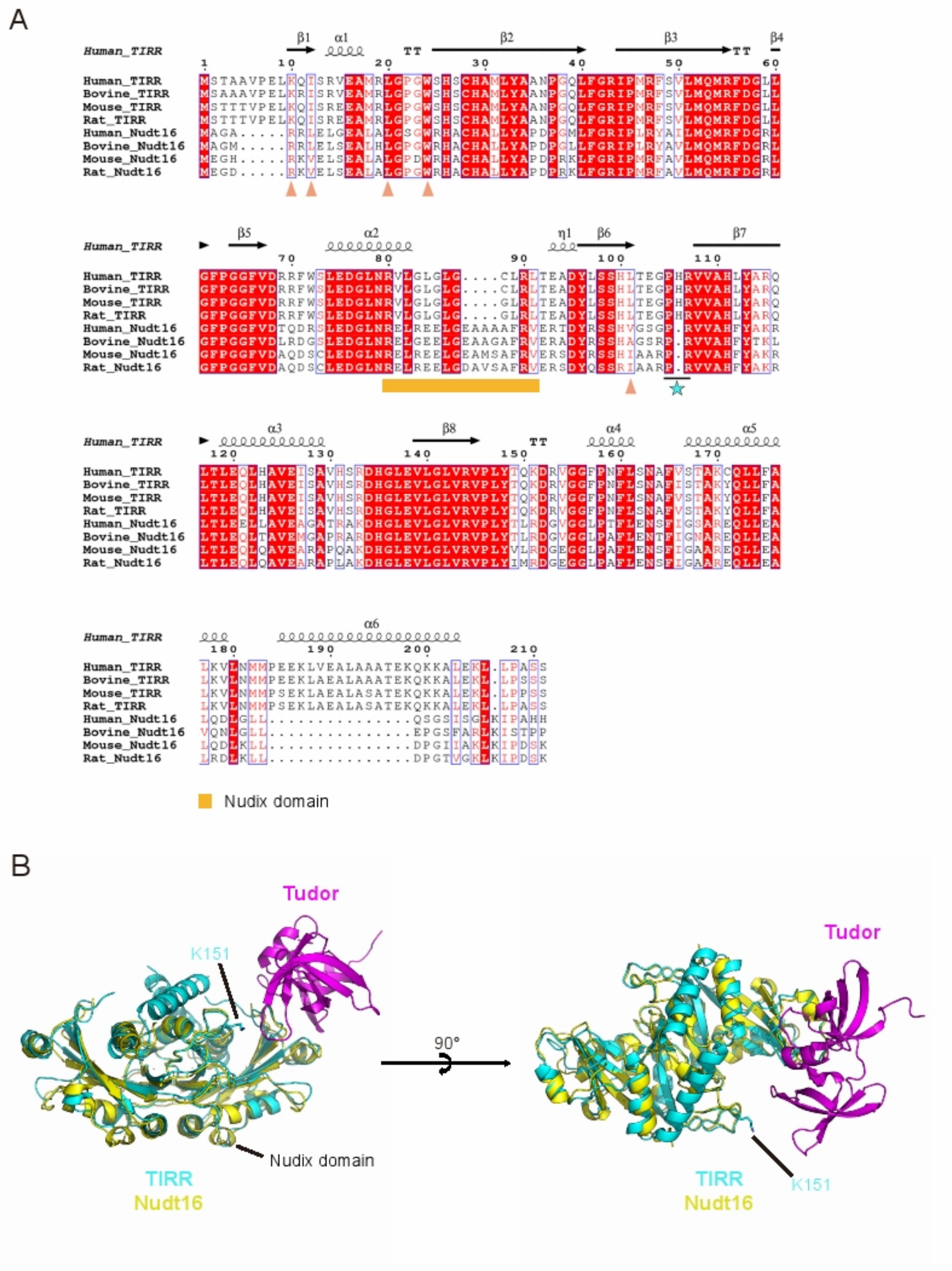

Supplementary Fig. 6. Sequence and structure comparison of TIRR and Nudt16. **A.** Multiple sequence alignment of TIRR and Nudt16 from human, bovine, mouse, rat is displayed. The residues participating in the 53BP1 interaction of TIRR are labeled by the orange triangle and the histidine insertion in L8-loop is highlighted by a cyan star. Highlighted in yellow represents the NUDIX domain of Nud16. **B.**Superimposition of the overall structure of TIRR (this study, cyan) and Nudt16(PDB 3COU, yellow). Highlight in the stick is TIRR residue K151.

Supplementary Figure 7

A

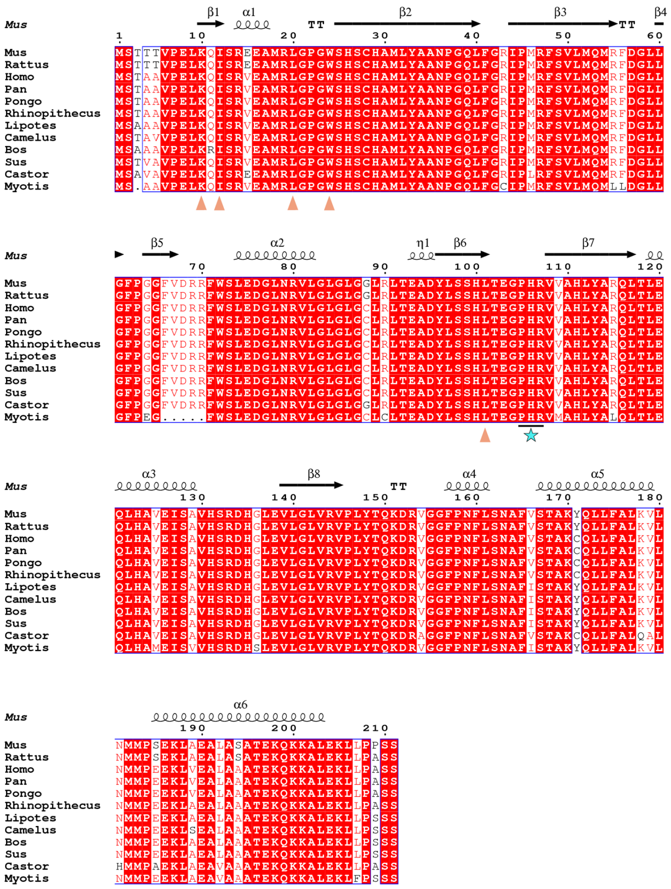

B

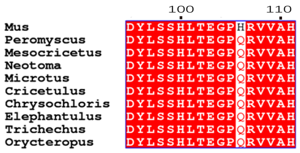

Supplementary Fig. 7. The importance and evolution of His106 in TIRR. A. Multiple sequence alignment of TIRR from different species. The conserved residues used for 53BP1 Tudor interaction are indicated in the orange triangle, with a conserved histidine insertion in L8-loop highlighted in a cyan star. **B.**Sequence alignment of TIRR from some species that contain an insertion of glutamine (^Q) in the L8-loop.

Supplementary Figure 8

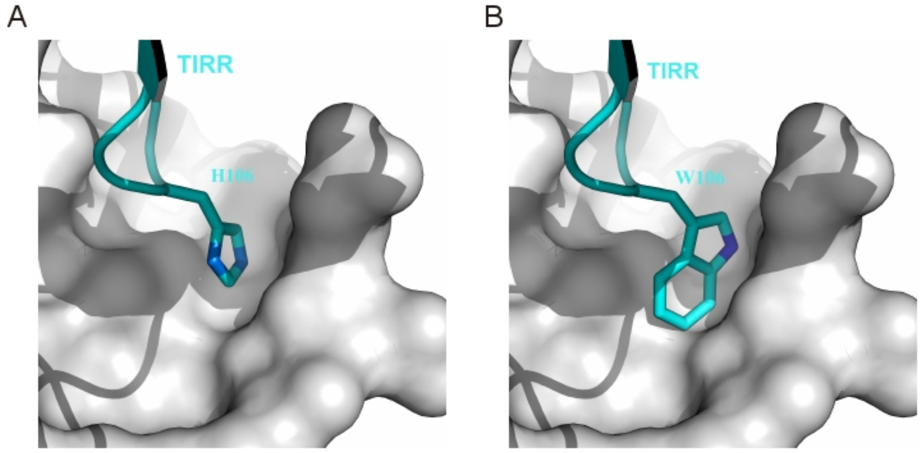

C

Nudt16	Binding affinity(K_d,μM)
WT	ND*
^H105	7.1
^W105	3.6

*ND means Not Detected

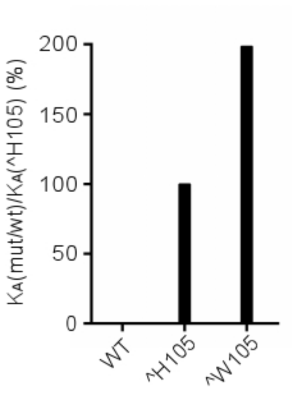

Supplementary Fig. 8. Effect of the histidine or tryptophan insertion in L8-loop of Nudt16. (A,B). The interaction environment of His106 and substituted Trp106 of TIRR with 53BP1 Tudor.**C.**ITC results of the titration of 53BP1 Tudor to different Nudt16 insertion mutants.

Supplementary Figure 9

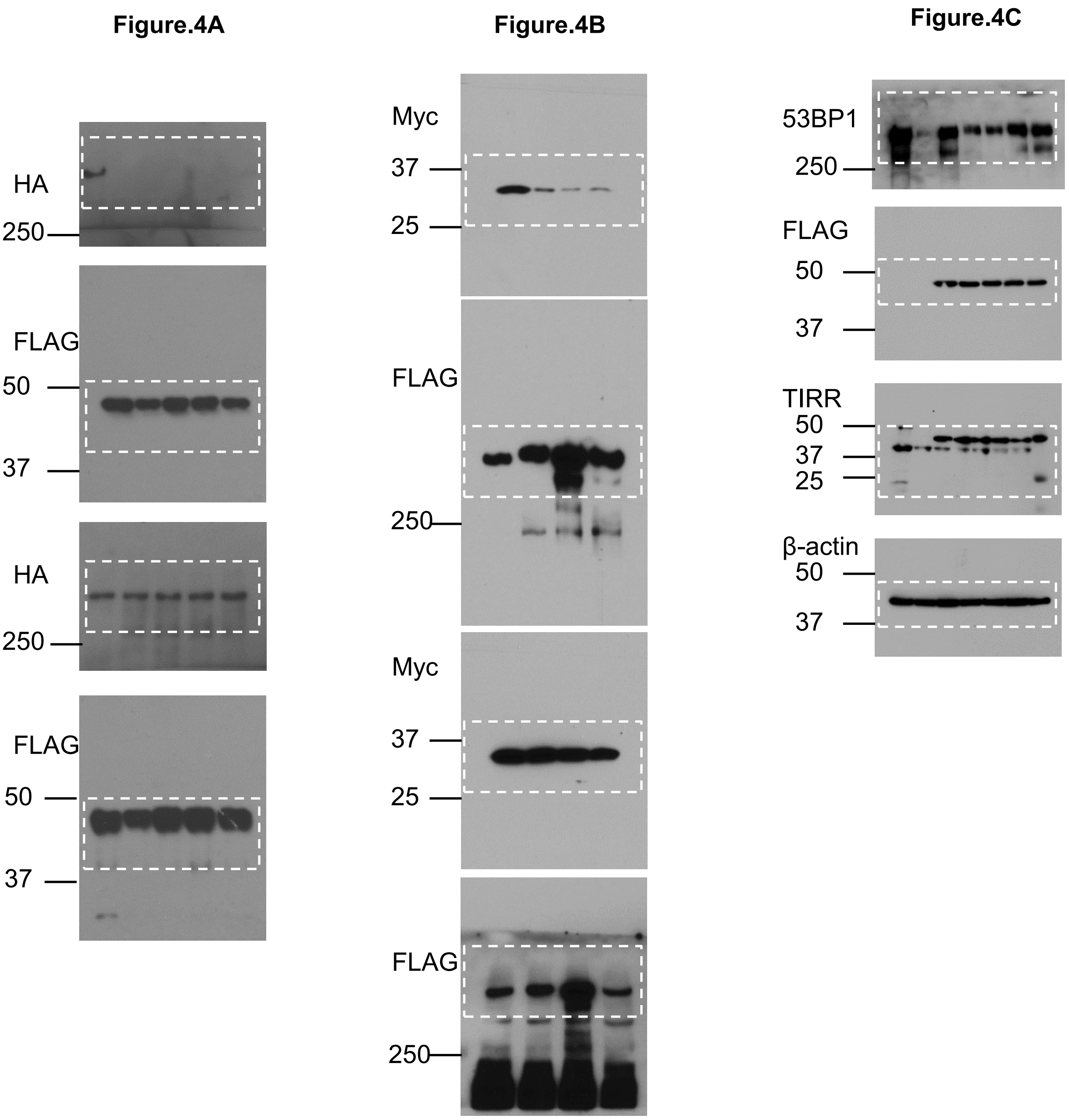

Supplementary Fig. 9. Original images of western blots shown in this study.

Supplementary Table 1. List of primers used in TIRR construct

Primer name	Sequence (5' - 3')
Human TIRR6 F	GGAATTCCATATGGTTCCGGAGCTGAAGCAG
Human TIRR211 R	CCGCTCGAGAGAGGAGGCCGGGAGCAACTTCTC
Mouse TIRR6 F	GGAATTCCATATGGTTCCGGAGCTGAAACAGATCAG
Mouse TIRR211 R	CCGCTCGAGTCAGGATGAGGGCGGGAGCAG
Mouse TIRRK10E F	CCGGAGCTGGAACAGATCAGTCGGGAGGAAG
Mouse TIRRK10E R	GACTGATCTGTTCCAGCTCCGGAACCATATGGCC
Mouse TIRRW24A F	GGGCCCGGCGCGAGTCATTCATGCCACGCCATG
Mouse TIRRW24A R	CATGAATGACTCGCGCCGGGCCCCAAGCGCATTGCTTC
Mouse TIRRR107A F	GGTCCACACGCGGTGGTGGCACATCTGTACGCACG
Mouse TIRRR107A R	GTGCCACCACCGCGTGTGGACCCTCAGTCAGGTG
Mouse TIRRP105A F	CTGAGGGTGCGCACCGTGTGGTGGCACATCTGTAC
Mouse TIRRP105A R	CACCACACGGTGCGCACCCTCAGTCAGGTGTGAACTC
Mouse TIRRI12G F	CTGAAACAGGGCAGTCGGGAGGAAGCAATGCGCTTG
Mouse TIRRI12G R	CCTCCCGACTGCCCTGTTTCAGCTCCGGAACCATATG
Mouse TIRRI12A F	CTGAAACAGGCGAGTCGGGAGGAAGCAATGCGCTTG
Mouse TIRRI12A R	CCTCCCGACTCGCCTGTTTCAGCTCCGGAACCATATG
Mouse TIRRL20G F	CAATGCGCGGCGGGCCCGGCTGGAGTCATTCATG
Mouse TIRRL20G R	CAGCCGGGCCCGCCGCGCATTGCTTCCTCCCGACTGAT
Mouse TIRRL20A F	CAATGCGC GCG GGGCCCGGCTGGAGTCATTCATG
Mouse TIRRL20A R	CAGCCGGGCCCCGCGCGCATTGCTTCCTCCCGACTGAT
Mouse TIRRH106A F	GAGGGTCCAGCGCGTGTGGTGGCACATCTGTAC
Mouse TIRRH106A R	GCCACCACACGCGCTGGACCCTCAGTCAGGTGTG
Mouse TIRRH106Δ F	GAGGGTCCACGTGTGGTGGCACATCTGTAC
Mouse TIRRH106Δ R	GCCACCACACGTGGACCCTCAGTCAGGTGTG
Mouse TIRRH106Y F	GAGGGTCCATATCGTGTGGTGGCACATCTGTAC
Mouse TIRRH106Y R	GCCACCACACGATATGGACCCTCAGTCAGGTGTG
Mouse TIRRH106W F	GAGGGTCCATGGCGTGTGGTGGCACATCTGTAC
MouseTIRRH106W R	GCCACCACACGCCATGGACCCTCAGTCAGGTGTG
Mouse TIRRH106R F	GAGGGTCCACGTCGTGTGGTGGCACATCTGTAC
Mouse TIRRH106R R	GCCACCACACGACGTGGACCCTCAGTCAGGTGTG
Mouse TIRRH106K F	GAGGGTCCAAAACGTGTGGTGGCACATCTGTAC
Mouse TIRRH106K R	GCCACCACACGTTTTGGACCCTCAGTCAGGTGTG
Mouse TIRRH106Q F	GAGGGTCCACAGCGTGTGGTGGCACATCTGTAC
Mouse TIRRH106Q R	GCCACCACACGCTGTGGACCCTCAGTCAGGTGTG
Human TIRRK10A F	CGGTTCCGGAGCTGGCGCAGATCAGCCGG
Human TIRRK10A R	CCGGCTGATCTGCGCCAGCTCCGGAACCG
Human TIRRW24A F	CCTAGGGCCGGGCGCGAGCCACTCGTGC
Human TIRRW24A R	GCACGAGTGGCTCGCGCCCGGCCCTAGG
Human TIRRH106A F	GACCGAGGGCCCAGCCCGCGTCGTGGC
Human TIRRH106A R	GCCACGACGCGGGCTGGGCCCTCGGTC
Human TIRRR107A F	CGAGGGCCCACACGCCGTCGTGGCGCACC
Human TIRRR107A R	GGTGCGCCACGACGGCGTGTGGGCCCTCG

Supplementary Table 2. List of primers used in 53BP1 construct

Primer name	Sequence (5' - 3')
53BP1 1484 F	GGAATTCCATATGAATAGCTTTGTAGGGCTCCGTGTTG
53BP1 1603 R	CGGGATCCTCAGCCAAGCCCATACTGCTCTCTCAG
53BP1 1631 R	CGGGATCCTCAACTGCGCCGTTTCCGCTTCC
53BP1W1495A F	GTTGTAGCCAAGGCGTCATCCAATGGCTACTTTTACTC
53BP1W1495A R	GCCATTGGATGACGCCTTGGCTACAACACGGAGC
53BP1Y1500A F	CATCCAATGGCGCGTTTTACTCTGGGAAAATCACAC
53BP1Y1500A R	CAGAGTAAAACGCGCCATTGGATGACCACTTGGC
53BP1Y1502A F	CAATGGCTACTTTGCGTCTGGGAAAATCACACGAGATG
53BP1Y1502A R	GATTTTCCCAGACGCAAAGTAGCCATTGGATGACCAC
*53BP1Y1502A F	GTCATCCAATGGCTACTTTGCCTCTGGGAAAATCACACG
*53BP1Y1502A R	CGTGTGATTTTCCCAGAGGCAAAGTAGCCATTGGATGAC
53BP1D1521A F	GCTCTTTGATGCGGGGTACGAATGTGATGTGTTG
53BP1D1521A R	CATTCGTACCCCGCATCAAAGAGCAATTTATACTTCCC
53BP1D1521R F	GCTCTTTGATCGTGGGTACGAATGTGATGTGTTG
53BP1D1521R R	CATTCGTACCCACGATCAAAGAGCAATTTATACTTCCC
*53BP1D1521R F	GGAAGTATAAATTGCTCTTTGATCGTGGGTACGAATGTGATG TGTTGG
*53BP1D1521R R	CCAACACATCACATTCGTACCCACGATCAAAGAGCAATTTAT ACTTCC
*53BP1Y1523A F	GCTCTTTGATGATGGGGCCGAATGTGATGTGTTGGG
*53BP1Y1523A R	CCCAACACATCACATTCGGCCCCATCATCAAAGAGC
53BP1Y1523A F	GATGATGGGGCGGAATGTGATGTGTTGGGCAAAG
53BP1Y1523A R	CATCACATTCCGCCCCATCATCAAAGAGCAATTTATAC
53BP1Y1523S F	GATGATGGGAGCGAATGTGATGTGTTGGGCAAAG
53BP1Y1523S R	CATCACATTCGCTCCCATCATCAAAGAGCAATTTATAC
53BP1L1547A F	GAAGTGACGGCCGCGTCGGAGGATGAGTATTTCAGTG
53BP1L1547A R	CTCATCCTCCGACGCGGCCGTCACTTCAGTGTCCAG
53BP1L1547G F	GAAGTGACGGCCGGCTCGGAGGATGAGTATTTCAGTG
53BP1L1547G R	CTCATCCTCCGAGCCGGCCGTCACTTCAGTGTCCAG
53BP1F1553A F	GAGGATGAGTATGCGAGTGCAGGAGTGGTGAAAGGAC
53BP1F1553A R	CACTCCTGCACTCGCATACTCATCCTCCGAGAGGGC

*These primers are used for *in vivo* study

Supplementary Table 3. List of primers used in Nudt16 construct

Primer name	Sequence (5' - 3')
Human NUDT16 F	GGAATTCCATATGGCCGGAGCCCGCAGGC
Human NUDT16 R	CGGGATCCCTAGTGATGAGCTGGAATCTTAAGGCCTG
NUDT16 ^H105 F	GTCAGGGCCACATCGCGTTGTGGCCCACTTCTATG
NUDT16 ^H105 R	CCACAACGCGATGTGGCCCTGACCCGACGTGG
NUDT16 ^W105 F	GTCAGGGCCATGGCGCGTTGTGGCCCACTTCTATG
NUDT16 ^W105 R	CCACAACGCGCCATGGCCCTGACCCGACGTGG
NUDT16 R5K F	GCCGGAGCCAAAAGGCTGGAGCTAGGCGAGGC
NUDT16 R5K R	GCTCCAGCCTTTTGGCTCCGGCCATATGGCCCTGA
NUDT16 L7I F	GAGCCAAAAGGATTGAGCTAGGCGAGGCCCTGGCG
NUDT16 L7I R	CCTCGCCTAGCTCAATCCTTTTGGCTCCGGCCATATG
NUDT16 V100L F	GCAGCTCCCACCTGGGGTCAGGGCCACATCGCGTTG
NUDT16 V100L R	GGCCCTGACCCCAGGTGGGAGCTGCGGTAGTCAGTG

Supplementary Table 4. List of antibodies.

Antibody	Company (Cat. No.)	Source	Application	Dilution
anti-HA	Invitrogen (26183)	Mouse monoclonal	Immunoblot	1:1000
anti-Flag M2	Sigma (F3165)	Mouse monoclonal	Immunoblot<br>Immunofluorescence	1: 4000<br>1: 10000
anti-RPA2(9H8)	Abcam (ab2175)	Mouse monoclonal	Immunofluorescence	1: 200
anti-β-actin (AC-15)	Sigma (A5441)	Mouse monoclonal	Immunoblot	1: 10000
anti-53BP1	Cell Signaling Technology (4937s)	Rabbit polyclonal	Immunoblot	1: 1000
anti-53BP1	In house	Rabbit polyclonal	Immunoblot<br>Immunofluorescence	1: 1000<br>1: 4000
anti-Myc (9E10)	Santa Cruz Biotechnology (sc-40)	Mouse monoclonal	Immunoblot<br>Immunofluorescence	1: 500<br>1: 1000
anti-γH2AX (JBW301)	Millipore Core (05 - 636)	Mouse monoclonal	Immunofluorescence	1: 400
anti-RIF1	Bethyl Laboratories (A300-568A)	Rabbit polyclonal	Immunofluorescence	1:500
anti-TIRR	In house	Rabbit polyclonal	Immunoblot	1: 1000

Supplementary References

1. Botuyan, M. V. et al. Structural Basis for the Methylation State-Specific Recognition of Histone H4-K20 by 53BP1 and Crb2 in DNA Repair. Cell 127, 1361–1373 (2006).
